# Coexistence of T-Cell Lymphoblastic Lymphoma and Ichthyosis Vulgaris: A Case Report

**DOI:** 10.1155/crom/5023552

**Published:** 2025-01-29

**Authors:** Şule Çalışkan Kamış, Begül Yağcı

**Affiliations:** Department of Pediatric Hematology and Oncology, Adana Faculty of Medicine, Adana City Education and Research Hospital, University of Health Sciences, Adana, Turkey

**Keywords:** case report, ichthyosis, lymphoma, whole-exome sequencing

## Abstract

Ichthyosis vulgaris (IV) is an inherited disorder characterized by the scaling of the skin. It is caused by mutations in the filaggrin gene. IV is a reactive skin manifestation that may be associated with malignant hematological disease. Its association with neoplastic diseases such as Hodgkin lymphoma, anaplastic large-cell lymphoma, and mycosis fungoides has been reported. T-cell non-Hodgkin lymphoma (T-NHL) with ichthyosis has been rarely reported in the literature. Here, we report a case of T-cell lymphoma with congenital IV caused by a desmoglein 1 (DSG1) gene mutation associated with hyper-IgE syndrome (HIES). A 7-year-old male patient with a diagnosis of congenital IV had a biopsy performed at an external center due to multiple lymphadenopathies, which revealed T-cell lymphoblastic lymphoma. A homozygous variant in the DSG1 gene was detected through whole-exome sequencing. The diagnosis of HIES was confirmed through clinical evaluation, including elevated serum IgE levels and associated symptoms. Skin findings, growth retardation, and HIES overlap with Online Mendelian Inheritance in Man (OMIM) #615508, and parental carrier status was confirmed. The association between ichthyosis and lymphoma was determined based on the presence of lymphoma in a patient with congenital ichthyosis and the identification of a genetic mutation in DSG1. In conclusion, the coexistence of lymphoma and IV is rare. The mechanisms of their formation are different, and they can occur independently. Rare genetic syndromes or inherited diseases can cause different health problems, such as lymphoma and ichthyosis, to occur together.

## 1. Introduction

Ichthyosis vulgaris (IV) is an inherited disease characterized by excessive scaling of the skin caused by a mutation in the filaggrin gene [1]. Netherton syndrome (Online Mendelian Inheritance in Man (OMIM) #256500) is a disease with an increased risk of both immunodeficiency and cancer. It is inherited in an autosomal recessive manner [2]. Morizane et al. reported ichthyosis in patients with anaplastic large-cell lymphoma and mycosis fungoides [3]. A pathogenic variant in the SPINK5 gene has been associated with Netherton syndrome [4]. Desmoglein 1 (DSG1) is involved in cell adhesion, and it is found in the upper layers of the epidermis. DSG1 participates in forming structures called desmosomes, which help maintain the structure and integrity of the skin [5, 6]. Netherton syndrome can also be associated with B-cell immunodeficiencies [7]. Elevated serum immunoglobulin E (IgE) levels are seen in Netherton syndrome [8]. There are rare cases suggesting a relationship between ichthyosis and lymphoma, particularly congenital IV. However, this association is extremely rare, and individuals with ichthyosis generally have a low risk of developing lymphoma [9, 10]. We report a case of T-cell lymphoma with congenital IV caused by a DSG1 mutation associated with hyper-IgE syndrome (HIES).

## 2. Case Report

**Figure 1:**
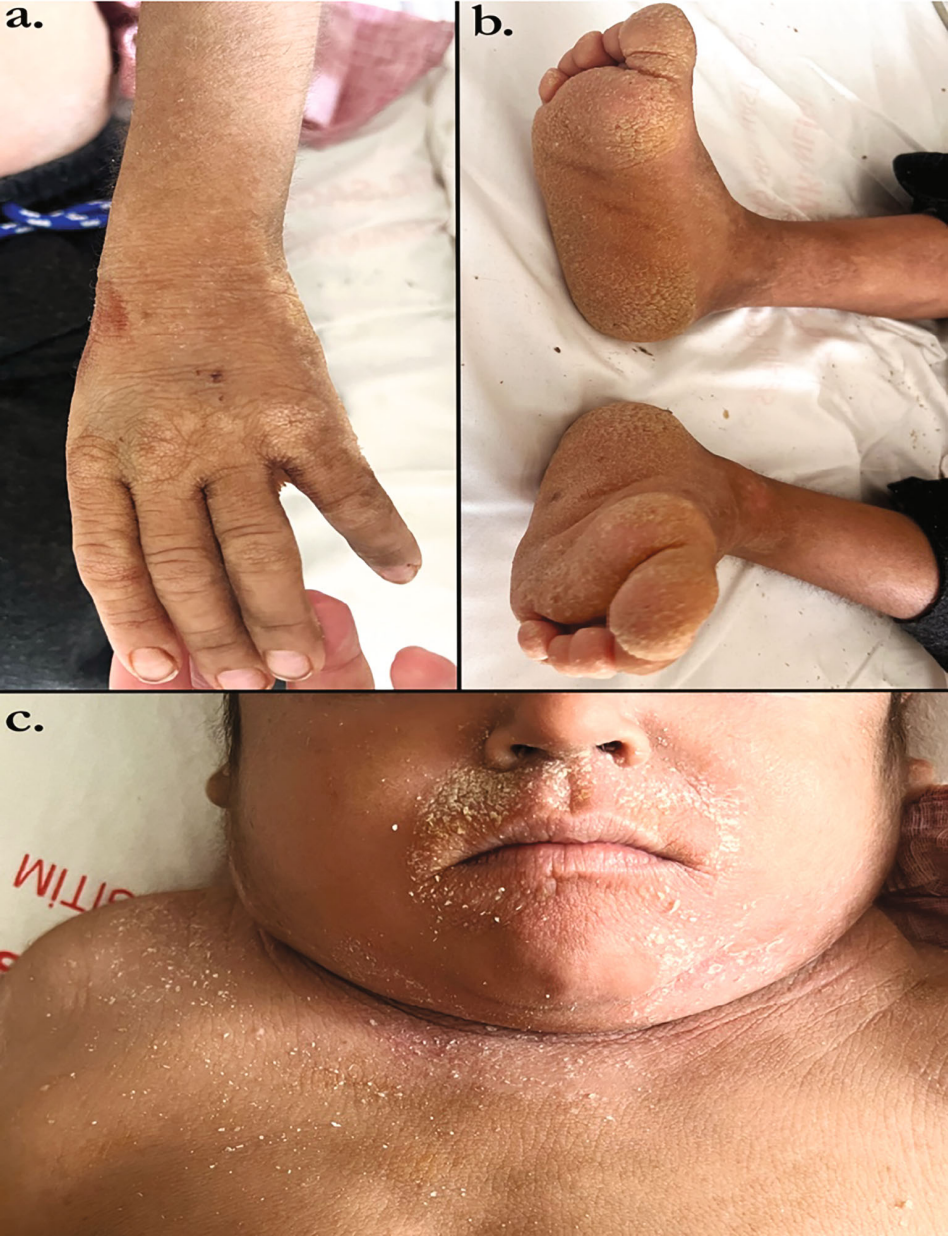
(a) Ichthyosis hand signs. (b) Ichthyosis foot signs. (c) Ichthyosis perioral findings.

A 7-year-old male patient with a diagnosis of congenital IV had a biopsy performed at an external center due to multiple lymphadenopathies (Figure 1). T-cell lymphoblastic lymphoma was detected as a result of the biopsy. The patient was referred to our hospital for further examination and treatment. He was admitted to the Pediatric Hematology and Oncology Clinic. A thorax computed tomography (CT) scan was performed due to the detection of pleural effusion. Hepatosplenomegaly was identified via abdominal ultrasound. In the pelvis and right lower quadrant, multiple intramesenteric lymph nodes with a heterogeneous conglomerate structure, the largest of which measured 28 × 23 mm, were observed. Thorax CT showed mosaic attenuation in the lungs, with a few mediastinal lymph nodes reaching pathological dimensions. Abdomen CT revealed an enlarged liver with numerous conglomerate lymph nodes in the periportal, paraaortic, and inter-aortacaval regions, as well as in the pelvis and bilateral inguinal areas. Morphological and immunohistochemical evaluation of the bone marrow biopsy showed high levels of CD2, CD3, CD4, CD5, and CD34, with a moderately dense, blastic lymphocytic population and a low positive reaction to CD8 and TdT. These findings were consistent with leukemic infiltration. The patient was evaluated by dermatology and genetics departments, and a homozygous variant in the DSG1 gene was detected through whole-exome sequencing. The diagnosis of HIES was made based on clinical criteria, including elevated serum IgE levels and other related symptoms. The patient's skin findings, growth retardation, and elevated IgE levels matched the characteristics of OMIM #615508, and parental carrier status was confirmed. A homozygous c.909G>C (p.W303C) variant in the DSG1 gene was identified, which is associated with “congenital erythroderma with palmoplantar keratoderma, hypotrichosis, and HIES (OMIM #615508). In addition, a heterozygous c.1521_1526delAAGCTC (p.S508_S509del) variant in the KRT10 gene, linked to ichthyosis, was detected. Both the patient's mother and father were found to carry the heterozygous c.909G>C (c.W303C) variant in the DSG1 gene.

## 3. Discussion

IV is characterized by dry, thick skin with a distinctive “fish-like” appearance and is considered a reactive skin manifestation associated with malignant hematological diseases such as Hodgkin lymphoma, anaplastic large-cell lymphoma, and mycosis fungoides. T-cell non-Hodgkin lymphoma associated with ichthyosis has been rarely reported in the literature [11–13]. Morizane et al. reported ichthyosis in patients with anaplastic large-cell lymphoma and mycosis fungoides [3]. Akpinar et al. reported a case of Hodgkin lymphoma associated with ichthyosis [14]. Katoa et al. reported a case of ichthyosis associated with anaplastic large-cell lymphoma [15]. The conclusion that ichthyosis is related to lymphoma rather than HIES is based on the clinical presentation of lymphoma in a patient with congenital ichthyosis and the detection of a specific genetic mutation in DSG1. The coexistence of lymphoma and ichthyosis is rare, and their development mechanisms are distinct. They can occur independently. Some rare genetic syndromes or inherited diseases may cause conditions like lymphoma and ichthyosis to present concurrently.

## 4. Conclusion

The coexistence of lymphoma and IV is rare, and their development mechanisms differ. Some rare genetic syndromes or inherited diseases may cause conditions like lymphoma and ichthyosis to manifest together.

## Data Availability

The data that support the findings of this study are available from the corresponding author upon reasonable request.
